# Uncovering the mortality burden of lung cancer with documented sleep apnea in the United States: a 22-year population-based study

**DOI:** 10.3389/fpubh.2026.1925388

**Published:** 2026-09-30

**Authors:** Lan Lin, Ping Shi, Qingyuan Li, Tong Feng, Ran Duan, Pengcheng Zheng

**Affiliations:** 1 Department of Nursing, The Second Affiliated Hospital of Chengdu Medical College (China National Nuclear Corporation 416 Hospital), Chengdu, China; 2 Department of Respiratory and Critical Care, Guangyuan Central Hospital, Guangyuan, China; 3 School of Clinical Medicine, Chengdu Medical College, Chengdu, China; 4 Department of Respiratory and Critical Care Medicine, The First Affiliated Hospital of Chengdu Medical College, Chengdu, China; 5 Laboratory of Geriatric Respiratory Diseases of Sichuan Higher Education Institute, Chengdu, China; 6 Department of Respiratory and Critical Care Medicine, Deyang People’s Hospital, Affiliated Hospital of Chengdu Medical College, Deyang, China; 7 Department of Oncology, The First Affiliated Hospital of Chengdu Medical College, Chengdu, China; 8 Key Laboratory of Intelligent Medical Care and Elderly Health Management, Chengdu, China

**Keywords:** age-adjusted mortality rate, CDC WONDER, Joinpoint regression, lung cancer, mortality, sleep apnea

## Abstract

**Background:**

Lung cancer remains the leading cause of cancer-related mortality worldwide, and sleep apnea may identify a clinically vulnerable subgroup of patients with lung cancer. However, population-level mortality patterns involving lung cancer with documented sleep apnea remain unclear. This study examined long-term trends, disparities, geographic variation, socioeconomic patterns, and future projections of related mortality in the United States.

**Methods:**

We conducted a retrospective population-based analysis using CDC WONDER Multiple Cause of Death data from 1999 to 2020. Deaths were included when lung cancer was recorded as the underlying cause of death and sleep apnea as a contributing cause. Age-adjusted mortality rates (AAMRs) were calculated per 100,000 population. Temporal trends were assessed using Joinpoint regression, historical AAMRs were projected with ARIMA models, out-of-sample performance was evaluated against final 2021–2024 CDC WONDER data, and state-level Sociodemographic Index and frontier analyses were performed.

**Results:**

A total of 4,339 deaths involving lung cancer with documented sleep apnea were identified. The overall AAMR was 0.05 per 100,000 population. Mortality was concentrated predominantly among adults aged 65 years and older and was higher among men, White and non-Hispanic individuals, residents of nonmetropolitan areas, and those living in the Midwest and West. The AAMR increased from 0.011 in 1999 to 0.112 in 2020, with a significant annual percent change of 9.398%. The ARIMA model projected an increase to 0.160 per 100,000 by 2030, but it consistently overestimated observed AAMRs during 2021–2024 (MAPE 9.71%); therefore, these forecasts were interpreted as conditional and exploratory projections. State-level analyses showed marked geographic heterogeneity not fully explained by socioeconomic development.

**Conclusion:**

Mortality involving lung cancer and documented sleep apnea increased substantially over 22 years and showed clear demographic and geographic disparities, with a disproportionate burden among older adults, particularly those aged 65 years and above. The historical trend and exploratory forecasting results support continued attention to sleep health assessment and integrated respiratory-oncology care.

## Graphical abstract

**Figure:**
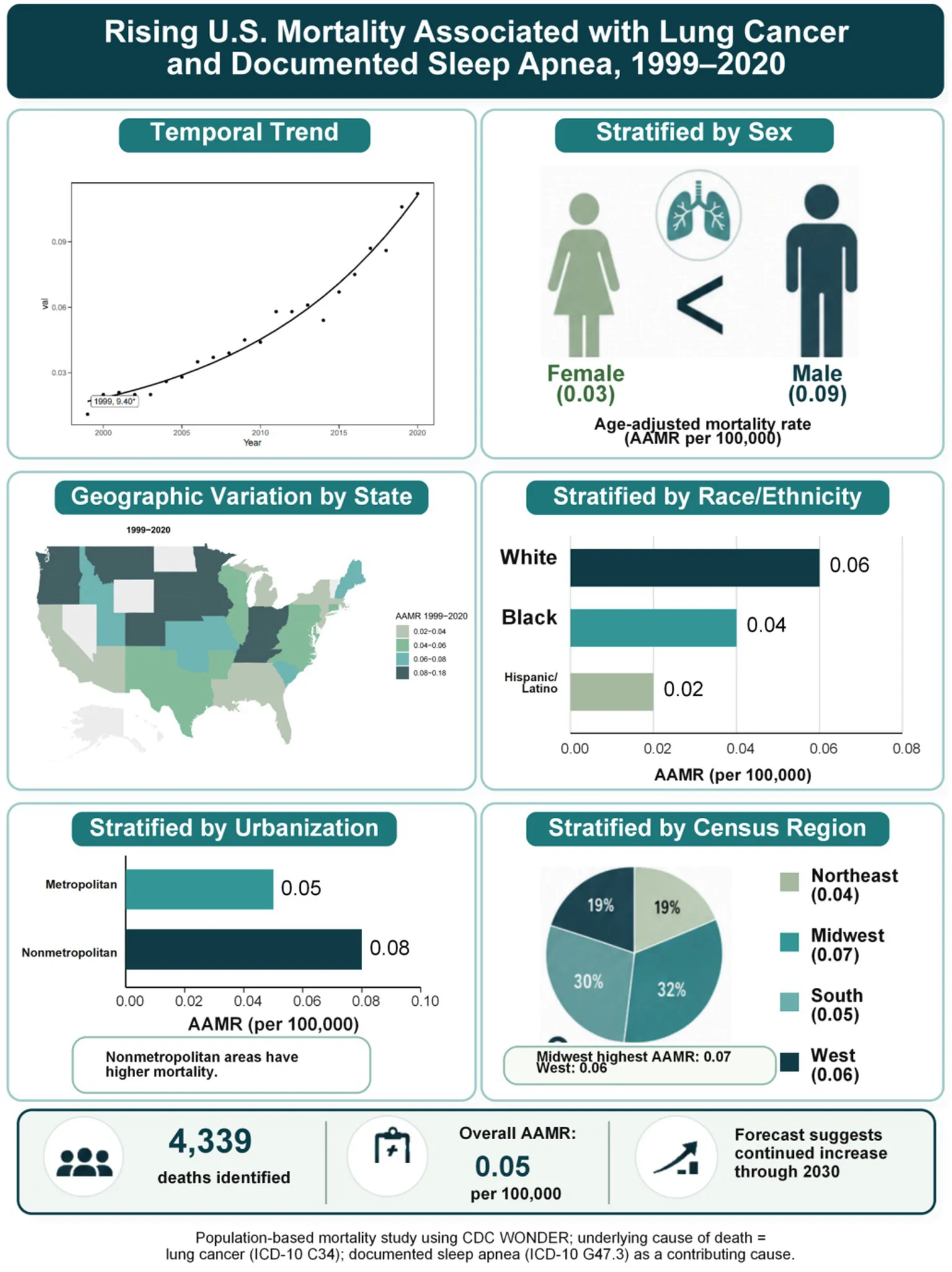

## Introduction

Lung cancer remains a major public health challenge and the leading cause of cancer-related mortality worldwide. In 2022, lung cancer accounted for approximately 2.5 million new cases and 1.8 million deaths globally, representing the highest mortality burden among all cancers (1). Although advances in early detection, targeted therapy, immunotherapy, and multidisciplinary care have improved outcomes for selected patients, lung cancer continues to impose a substantial population-level mortality burden. Identifying comorbid conditions that may characterize vulnerable lung cancer populations is therefore important for risk stratification, clinical surveillance, and public health planning.

Sleep apnea comprises a heterogeneous group of sleep-related breathing disorders, among which obstructive sleep apnea (OSA) is the most common and most extensively studied subtype. OSA is characterized by recurrent upper-airway obstruction during sleep, intermittent hypoxemia, sleep fragmentation, and sympathetic activation (2). Global estimates suggest that nearly 1 billion adults aged 30–69 years may have OSA, highlighting its substantial population-level burden (3). Beyond its effects on sleep quality, OSA has been linked to cardiovascular, metabolic, respiratory, and inflammatory disorders (4). These systemic consequences may be clinically relevant in individuals with lung cancer.

Most mechanistic evidence concerning the potential relationship between sleep apnea and cancer has focused on OSA. Chronic intermittent hypoxia, a hallmark of OSA, can induce oxidative stress, systemic inflammation, immune dysregulation, endothelial dysfunction, and activation of hypoxia-related signaling pathways (5). Experimental and translational studies further suggest that intermittent hypoxia may remodel the tumor microenvironment by promoting angiogenesis, epithelial–mesenchymal transition, tumor-associated macrophage polarization, cancer stemness, and immune escape (6). In lung cancer, these mechanisms may be clinically relevant because tumor progression and treatment response are strongly influenced by hypoxia-related pathways, inflammatory signaling, and the local immune microenvironment.

Epidemiological evidence regarding OSA and lung cancer has grown but remains inconsistent. A systematic review and meta-analysis reported a higher risk of lung cancer among individuals with OSA, particularly in studies with longer follow-up (7). Clinical studies have also suggested associations between nocturnal hypoxemia and lung cancer prevalence or incidence among patients evaluated for OSA (8–10), while OSA severity has been associated with increased cancer mortality or poorer survival in selected populations (11, 12). Conversely, other population-based studies have reported sex-specific or inverse associations between OSA and lung cancer incidence (13). Importantly, these studies primarily evaluated clinically diagnosed OSA and therefore differ from the present analysis, which identifies documented sleep apnea from death certificates using ICD-10 code G47.3. Accordingly, OSA-related epidemiological findings are discussed as contextual evidence rather than as direct evidence regarding the broader category of documented sleep apnea examined in this study.

Despite increasing attention to the OSA–lung cancer relationship, national mortality patterns involving both lung cancer and documented sleep apnea remain poorly characterized. Most previous studies have focused on cancer incidence, biological mechanisms, or selected clinical cohorts, whereas long-term temporal trends, demographic disparities, geographic variation, and socioeconomic gradients in mortality involving these coexisting conditions have not been systematically evaluated at the population level. Because death certificate data capture conditions documented as underlying or contributing causes of death, they provide an opportunity to describe the real-world mortality burden associated with lung cancer and documented sleep apnea, while acknowledging that such data cannot establish causality.

Therefore, using the CDC WONDER Multiple Cause of Death database, this study aimed to examine temporal trends and disparities in lung cancer and documented sleep apnea-related mortality in the United States from 1999 to 2020. Specifically, we assessed age-adjusted mortality rates, changes over time, demographic differences, geographic variation, and future mortality projections across the U. S. population to identify high-risk groups and inform targeted strategies for sleep-health assessment, respiratory care, and integrated cancer management.

## Methods

### Study design and data source

This retrospective, population-based mortality study used publicly available Multiple Cause of Death data from the Centers for Disease Control and Prevention Wide-ranging Online Data for Epidemiologic Research database (CDC WONDER). Mortality data were extracted for the United States from 1999 to 2020. The study period began in 1999 because cause-of-death coding in CDC WONDER has been based on the International Classification of Diseases, 10th Revision (ICD-10), since that year. This study was conducted and reported in accordance with the Strengthening the Reporting of Observational Studies in Epidemiology (STROBE) guideline (14).

Because CDC WONDER provides de-identified, aggregated, publicly available mortality data, this study did not involve identifiable human participants. Therefore, institutional review board approval and informed consent were not required.

### Study population and outcome definition

We included all deaths among US residents from 1999 to 2020 in which lung cancer was recorded as the underlying cause of death and sleep apnea was documented as a contributing cause of death on the death certificate. Lung cancer deaths were identified using ICD-10 code C34, corresponding to malignant neoplasm of the bronchus and lung. Sleep apnea was identified using ICD-10 code G47.3. This code encompasses obstructive, central, and unspecified sleep apnea; therefore, the available mortality data do not permit reliable identification of OSA as a distinct subtype. Deaths were included only when lung cancer was listed as the underlying cause of death and sleep apnea appeared in the multiple-cause-of-death fields as a contributing condition.

Eligible death records were identified from the CDC WONDER Multiple Cause of Death database using prespecified cause-of-death criteria. A total of 56,806,341 deaths among U. S. residents were recorded from 1999 to 2020. Of these, 3,380,830 deaths had lung cancer (ICD-10 code C34) recorded as the underlying cause of death. We further restricted the analysis to records in which documented sleep apnea was listed as a contributing cause of death in the multiple-cause-of-death fields. Ultimately, 4,339 deaths met both study criteria and were included in the final analysis. The study population selection process is summarized in Supplementary Figure 1.

The primary outcome was mortality with lung cancer as the underlying cause of death and documented sleep apnea as a contributing cause. Throughout the manuscript, the term “documented sleep apnea” refers specifically to sleep apnea recorded on death certificates under ICD-10 code G47.3. The term “OSA” is used only when referring to previous studies or mechanistic evidence that specifically investigated obstructive sleep apnea. Because CDC WONDER captures conditions recorded on death certificates rather than clinically adjudicated diagnoses, this analysis does not establish the specific sleep apnea subtype, nor does it infer that sleep apnea caused lung cancer or establish a causal relationship between sleep apnea and lung cancer mortality.

### Demographic and geographic variables

Mortality data were extracted by year of death, sex, age group, race, Hispanic origin, U. S. Census region, urbanization status, and state. Age groups were categorized as <1, 1–4, 5–14, 15–24, 25–34, 35–44, 45–54, 55–64, 65–74, 75–84, and ≥85 years. Race was classified as White, Black or African American, Asian or Pacific Islander, and American Indian or Alaska Native. Hispanic origin was classified as Hispanic or Latino and not Hispanic or Latino. U. S. Census regions were categorized as Northeast, Midwest, South, and West. Urbanization status was grouped as metropolitan and nonmetropolitan areas according to the CDC WONDER classification.

State-level analyses were performed to evaluate geographic differences in age-adjusted mortality rates. Estimates based on small death counts or suppressed values in CDC WONDER were treated as unavailable.

### Mortality measures

Death counts, crude mortality rates, and age-adjusted mortality rates (AAMRs) were extracted or calculated per 100,000 population. AAMRs were standardized to the 2000 U. S. standard population using the direct standardization method, allowing comparison across populations with different age structures (15). Corresponding 95% confidence intervals (CIs) were obtained from CDC WONDER when available. For subgroup analyses, estimates with suppressed or unreliable values were not interpreted as stable results.

### Temporal trend analysis

Temporal trends in annual AAMRs from 1999 to 2020 were assessed using Joinpoint regression. Joinpoint regression identifies statistically significant changes in mortality trends over time by fitting segmented log-linear models (16). Annual percent change (APC) and corresponding 95% CIs were estimated for each trend segment. Average annual percent change (AAPC) was calculated to summarize the overall trend across the entire study period. A trend was considered increasing or decreasing when the APC or AAPC was statistically different from zero. Statistical significance was defined as a two-sided *p* value <0.05.

### ARIMA forecasting

To project future mortality trends, autoregressive integrated moving average (ARIMA) models were fitted to annual national AAMRs from 1999 through 2020. Candidate models were screened using the auto.arima() function in R, with the Bayesian information criterion (BIC) specified as the model-selection criterion. The model with the lowest BIC was selected as the preferred model, resulting in an ARIMA(0,1,0) with drift.

Model adequacy was assessed using AIC, AICc, and BIC, agreement between observed and fitted values, and residual diagnostics. Residual distribution was evaluated using a Q-Q plot, while residual serial dependence was examined using the ACF, PACF, and Ljung–Box test at lag 4 (df = 4). Because the Ljung–Box test indicated modest residual autocorrelation, predictive performance was further assessed using out-of-sample validation. The model was fitted exclusively to the 1999–2020 observations, and its forecasts for 2021–2024 were compared with subsequently available final CDC WONDER observations for those years. Predictive performance was summarized using mean absolute error (MAE), root mean squared error (RMSE), and mean absolute percentage error (MAPE). The original ARIMA model was retained for generating the 2021–2030 projections. These forecasts were interpreted as conditional extrapolations of the historical 1999–2020 temporal pattern, rather than definitive predictions of future mortality. The subsequent 2021–2024 observations were used solely for out-of-sample validation and were not incorporated into the original model fitting.

### Sociodemographic index and frontier analysis

Annual state-specific Sociodemographic Index (SDI) values for 1999–2020 were obtained from the Global Burden of Disease Study 2021 (GBD 2021) Socio-Demographic Index dataset produced by the Institute for Health Metrics and Evaluation (IHME) (17). The published year-specific state SDI estimates were used directly and matched to mortality estimates for the same state and calendar year.

The frontier analysis was designed to estimate the lowest empirically achievable age-adjusted mortality rate (AAMR) at each level of SDI. The mortality dataset comprised 1,122 theoretical state-year observations (51 states/areas over 22 years). State-year observations with suppressed or unreliable AAMR estimates were excluded from frontier estimation. Specifically, 694 observations had suppressed AAMRs and 402 had unreliable AAMRs, leaving 26 state-year observations with reportable numeric AAMRs for the final frontier analysis. The nonlinear relationship between SDI and AAMR was estimated using locally estimated scatterplot smoothing (LOESS) with local polynomial regression, using a second-degree local polynomial (degree = 2) and a span of 0.75. The resulting lower boundary of the observed SDI–AAMR distribution was defined as the empirical best-performance frontier, representing the lowest mortality burden observed at comparable levels of socioeconomic development. For each state-year observation, the frontier gap was calculated as the observed AAMR minus the corresponding AAMR estimated from the fitted frontier at the same SDI level. Positive frontier gaps therefore indicated mortality burdens above the empirical best-performance frontier and represented potential room for improvement relative to the best observed performance at comparable levels of SDI. No arbitrary statistical significance threshold was used to define frontier observations; observations were evaluated according to their position and magnitude of deviation from the fitted frontier. Because this frontier was estimated nonparametrically from ecological state-year observations and was based on a limited number of reportable observations, the resulting frontier gaps were considered exploratory comparative indicators rather than causal effects, fixed mortality targets, or direct measures of healthcare efficiency.

### Statistical analysis

All analyses were conducted using R statistical software and Joinpoint Regression Program. Descriptive statistics were used to summarize death counts, population denominators, crude mortality rates, and AAMRs. Mortality patterns were compared across demographic and geographic subgroups. Statistical tests were two-sided, and *p* < 0.05 was considered statistically significant.

## Results

### Overall mortality burden and demographic disparities

From 1999 to 2020, 4,339 deaths involving lung cancer and documented sleep apnea were identified in the United States, with an overall age-adjusted mortality rate (AAMR) of 0.05 per 100,000 population (95% CI: 0.05–0.05; Table 1). Men had a higher mortality burden than women, with 2,930 deaths and an AAMR of 0.09 per 100,000 population, compared with 1,409 deaths and an AAMR of 0.03 among women. By race, White individuals accounted for most deaths and had the highest AAMR among major racial groups, whereas Asian or Pacific Islander individuals had the lowest AAMR. Non-Hispanic individuals had a higher AAMR than Hispanic individuals. Regional differences were also evident: the Midwest showed the highest AAMR, followed by the West, while nonmetropolitan areas had higher AAMR than metropolitan areas (Table 1).

**Table 1:** Demographic characteristics of deaths due to lung cancer and documented sleep apnea in the United States from 1999 to 2020.

Characteristic	Deaths/Population	AAMR (95% CI)
Overall	4339/6746356647	0.05(0.05,0.05)
Census region		
Midwest	1239/1466121214	0.07(0.07,0.07)
Northeast	591/1212994922	0.04(0.03,0.04)
South	1535/2497818081	0.04(0.04,0.05)
West	974/1569422430	0.06(0.05,0.06)
Sex		
Female	1409/3429003804	0.03(0.03,0.03)
Male	2930/3317352843	0.09(0.08,0.09)
Hispanic origin		
Hispanic or Latino	115/1077280338	0.02(0.01,0.02)
Not Hispanic or Latino	4219/5669076309	0.05(0.05,0.05)
Race		
American Indian or Alaska Native	24/88362592	0.04(0.03,0.07)
Asian or Pacific Islander	35/371914051	0.01(0.01,0.02)
Black or African American	327/919034937	0.04(0.04,0.05)
White	3953/5367045067	0.06(0.06,0.06)
Urbanization		
Metropolitan	3336/5739475649	0.05(0.05,0.05)
Nonmetropolitan	1003/1006871652	0.08(0.07,0.08)

### Geographic distribution

State-level mortality varied substantially across the United States. The geographic map showed higher AAMRs in several Midwestern, Mountain West, and Pacific Northwest states, whereas lower rates were observed in parts of the Northeast, South, and West Coast (Figure 1). State-level estimates confirmed this geographic heterogeneity, although several state estimates were unstable or suppressed because of small death counts (Supplementary Table 1).

**Figure 1:**
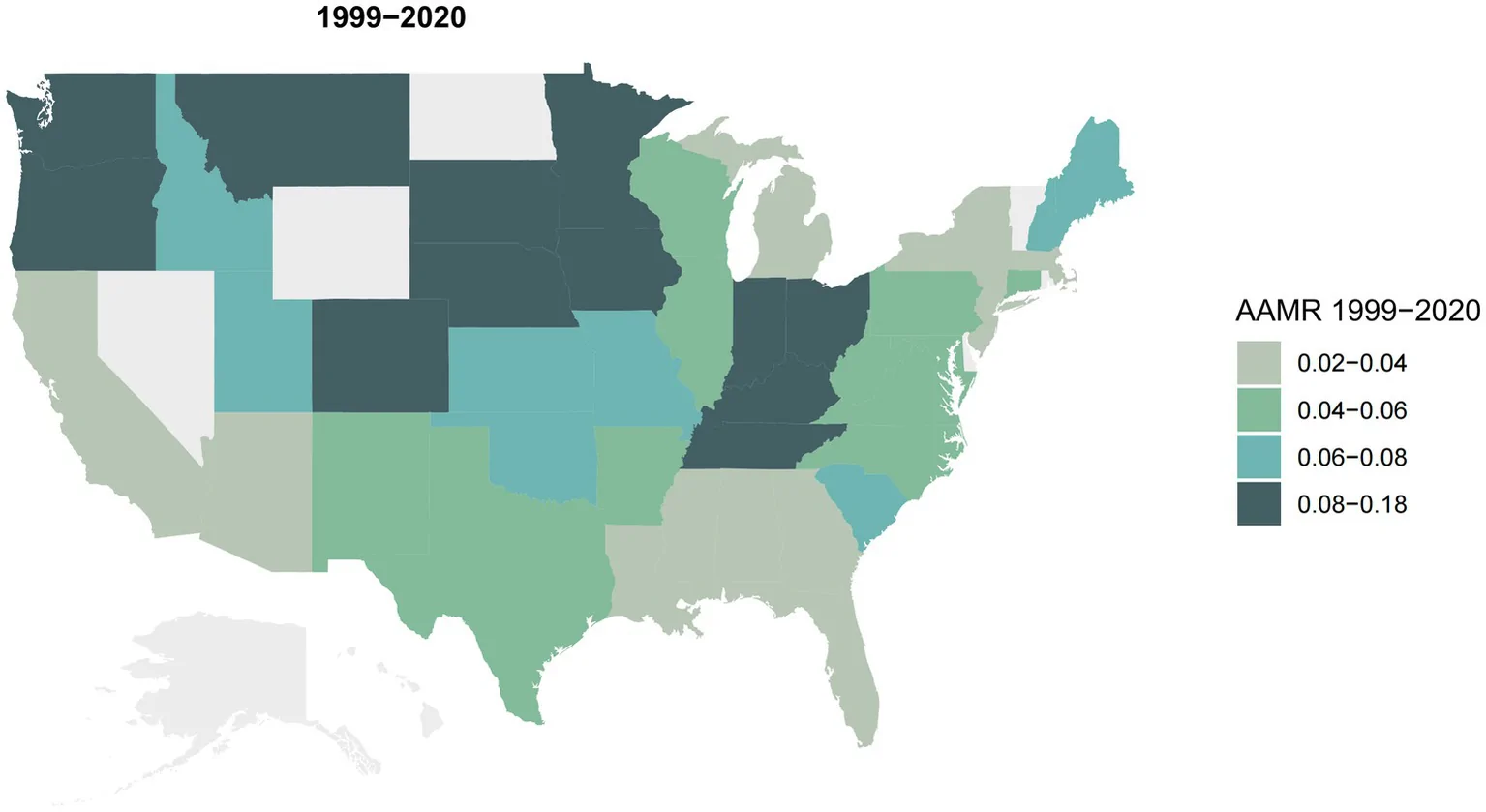
Geographic distribution of age-adjusted mortality rates for lung cancer and documented sleep apnea-related deaths in the United States, 1999–2020.

### Age- and sex-specific patterns

Age- and sex-specific analyses showed that mortality was concentrated mainly among older age groups, especially those aged 65 years and above. In 2020, men had higher mortality rates than women across comparable older age groups, with the highest rates observed among individuals aged 75–84 years (Supplementary Table 2; Supplementary Figure 2). Because CDC WONDER suppresses small-count age-by-sex cells, 3,913 of the 4,339 deaths were represented by reportable age-by-sex cells, whereas 426 deaths were contained in suppressed cells. For Supplementary Figure 3, the denominator for each year was the total number of deaths across age-by-sex cells with non-suppressed counts. Therefore, the annual bars represent the age composition of reportable deaths rather than the complete age distribution of all deaths, and suppressed deaths were not assigned to specific age categories.

### Temporal trend

Annual deaths and AAMRs increased markedly during the study period. Deaths rose from 37 in 1999 to 504 in 2020, while the overall AAMR increased from 0.011 to 0.112 per 100,000 population (Supplementary Table 3). This upward trend was consistent in the annual deaths plot and the temporal AAMR curve (Supplementary Figures 4, 5). Joinpoint regression demonstrated a significant increase in overall AAMR from 1999 to 2020, with an annual percent change of 9.398% (95% CI: 8.783–10.449; *p* < 0.001) and no joinpoints (Figure 2; Supplementary Table 4). Among men, one joinpoint was identified, with a rapid early increase from 1999 to 2001 followed by a sustained increase from 2001 to 2020 (Supplementary Table 4).

**Figure 2:**
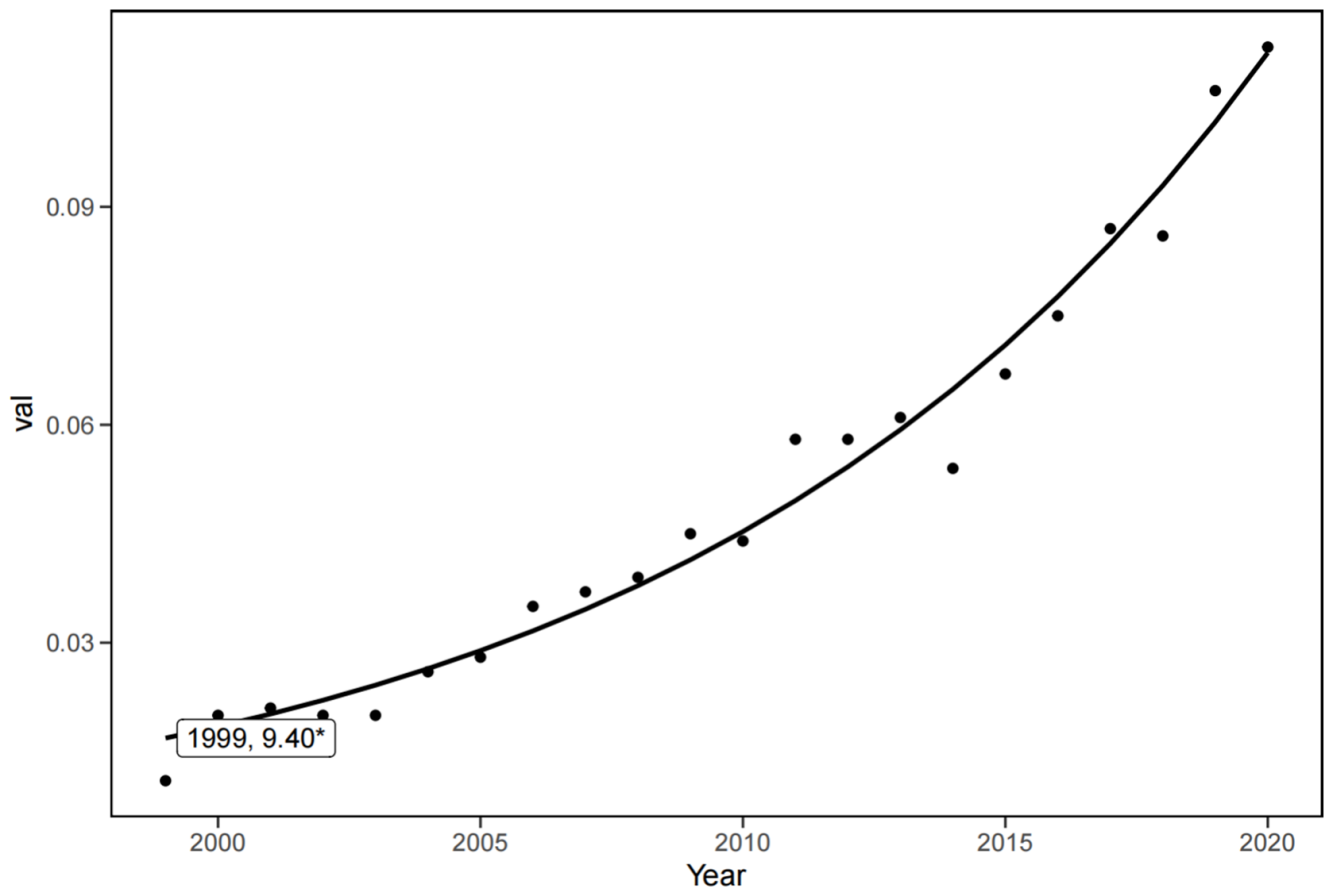
Joinpoint regression analysis of age-adjusted mortality trends for lung cancer and documented sleep apnea-related deaths, 1999–2020.

### Mortality projections and out-of-sample validation

The BIC-based auto.arima() procedure selected an ARIMA(0,1,0) model with drift for the 1999–2020 annual AAMR series. The selected model had an AIC of −150.108, AICc of −149.442, and BIC of −148.019, with strong agreement between observed and fitted AAMRs (*r* = 0.978, *p* < 0.001). Residual diagnostics indicated a modest departure from independence; the Ljung–Box test at lag 4 yielded Q = 9.915 (df = 4, *p* = 0.042).

To evaluate predictive performance, the original model was retained unchanged and its forecasts for 2021–2024 were compared with subsequently available final CDC WONDER observations. The model forecasted AAMRs of 0.117, 0.122, 0.126, and 0.131 per 100,000 for 2021, 2022, 2023, and 2024, respectively, whereas the corresponding observed AAMRs were 0.109, 0.113, 0.116, and 0.114 per 100,000. The resulting MAE was 0.0110 per 100,000, RMSE was 0.0116 per 100,000, and MAPE was 9.71%. The model correctly captured the overall upward direction of the mortality trend but showed systematic overprediction during the validation period, particularly in 2024.

The model-based projection subsequently extended from 2021 through 2030, with the point forecast increasing from 0.117 to 0.160 per 100,000 and prediction intervals widening over time (Figure 3; Supplementary Tables 5, 6). These projections were interpreted as conditional extrapolations of the historical mortality trajectory, rather than definitive predictions of future mortality.

**Figure 3:**
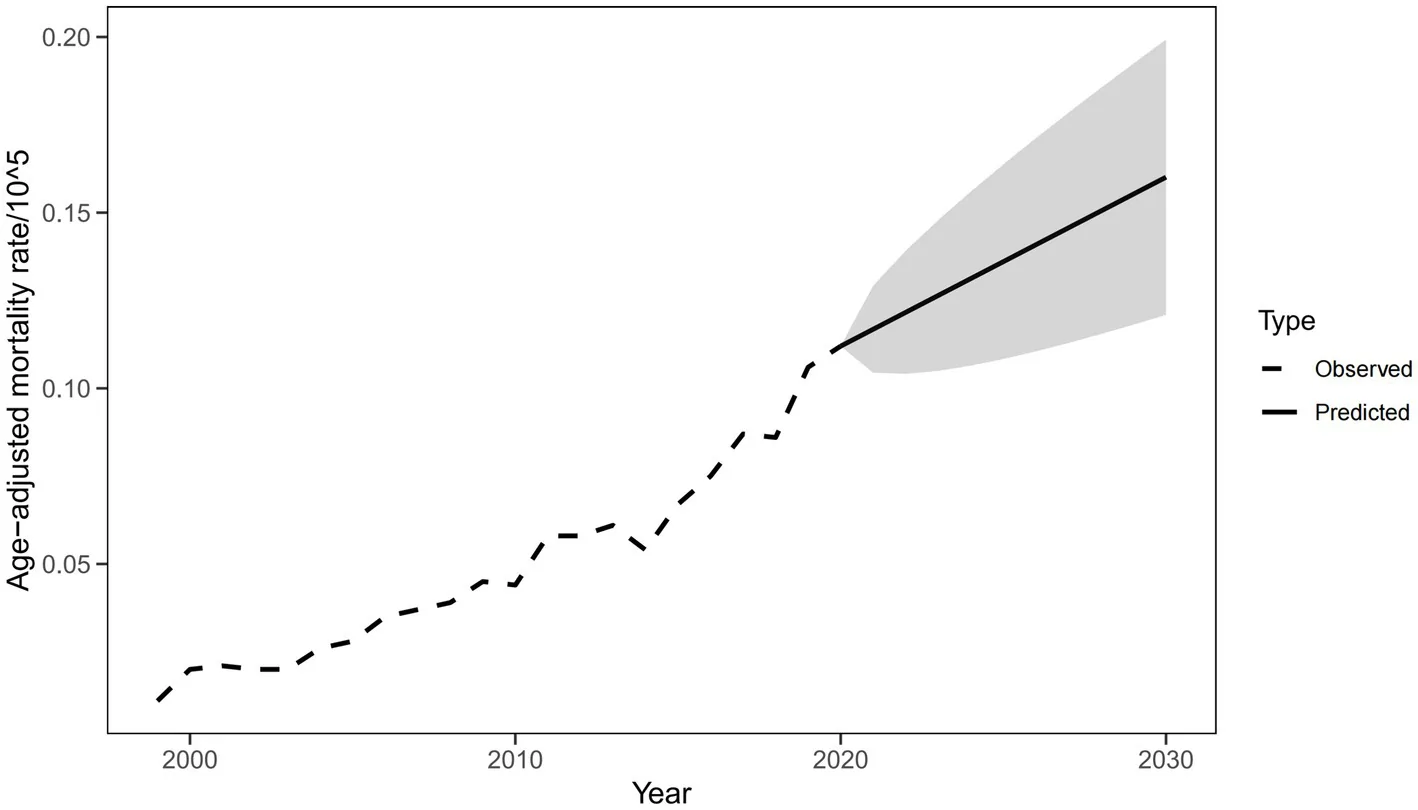
Observed and conditional ARIMA projections of age-adjusted mortality rates for lung cancer and documented sleep apnea-related deaths, 2021–2030.

### SDI and frontier analysis

Using annually matched state-specific SDI and AAMR estimates from 1999 to 2020, the SDI-based frontier analysis demonstrated a nonlinear relationship between socioeconomic development and mortality burden (Figure 4; Supplementary Figure 6). Of the 1,122 possible state-year observations, 694 were suppressed and 402 were classified as unreliable, leaving 26 state-year observations with reportable AAMRs for frontier estimation. The LOESS-based lower boundary represented the empirical best-performance frontier, corresponding to the lowest observed mortality burden at comparable SDI levels. Several state-year observations exhibited positive frontier gaps, indicating mortality burdens above the best observed performance at similar levels of socioeconomic development. These gaps were interpreted as potential areas for improvement rather than as causal effects or formal rankings of state performance.

**Figure 4:**
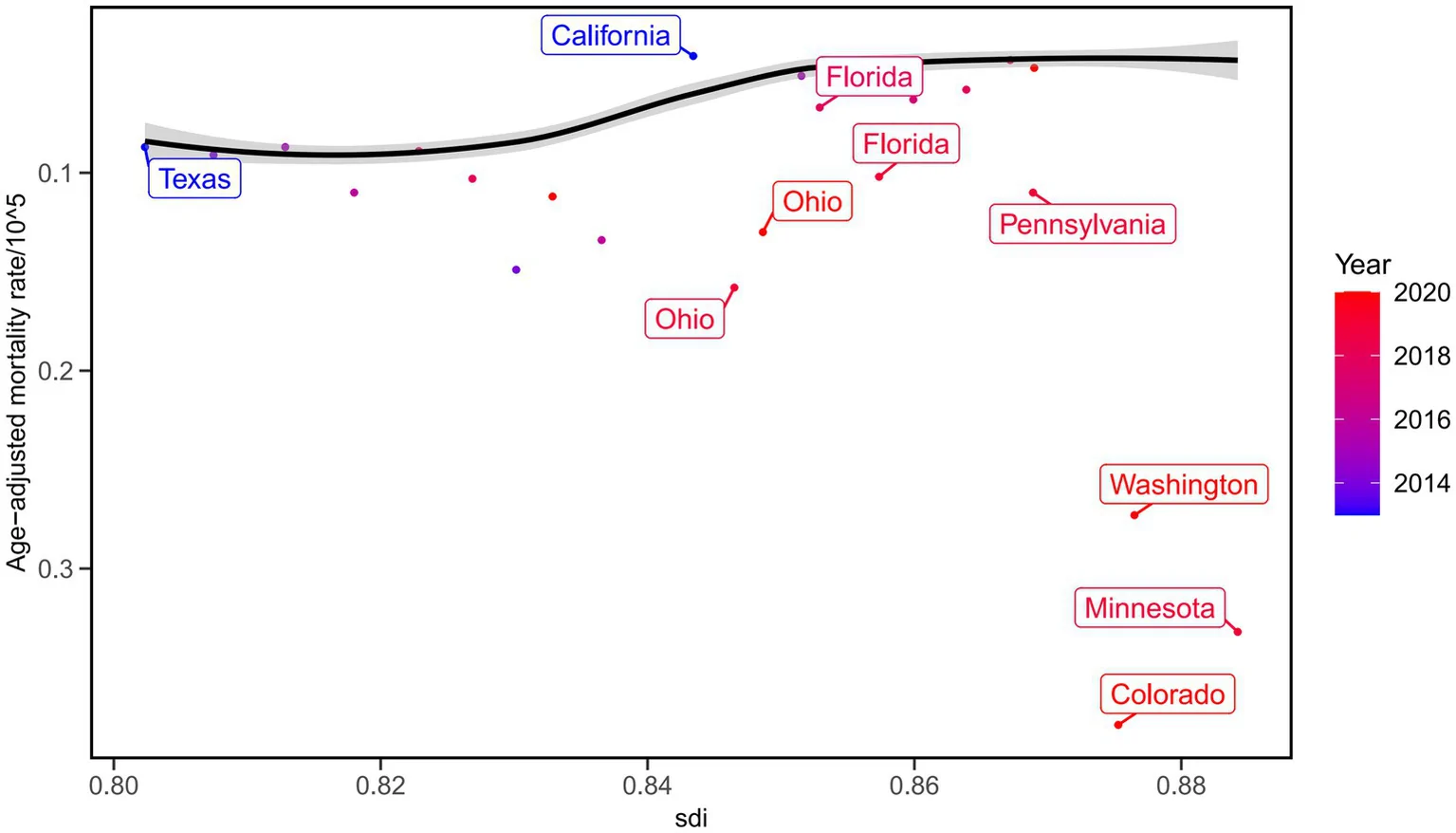
LOESS-based SDI frontier analysis of lung cancer and documented sleep apnea-related mortality across U. S. state-year observations.

The frontier findings were therefore considered exploratory and hypothesis-generating. Given the limited number of reportable state-year observations and the ecological nature of the analysis, the frontier should not be interpreted as a causal minimum mortality rate, a fixed policy target, or a definitive ranking of healthcare-system efficiency.

## Discussion

In this nationwide analysis of U. S. death certificates, 4,339 deaths were identified in which lung cancer was recorded as the underlying cause and sleep apnea was documented as a contributing condition between 1999 and 2020. Although the absolute mortality rate was low, the age-adjusted mortality rate (AAMR) increased approximately tenfold, from 0.011 to 0.112 per 100,000 population, with a sustained annual increase of 9.398% and no joinpoint in the overall trend. The burden was concentrated among adults aged 65 years or older and was substantially higher among men, White and non-Hispanic individuals, residents of the Midwest and West, and people living in nonmetropolitan areas. State-level variation was considerable and was not fully explained by the SDI. The forecasting analysis provided a conditional extrapolation of the historical trajectory rather than a definitive prediction; in out-of-sample validation, the model captured the overall upward direction but systematically overestimated observed AAMRs during 2021–2024. To our knowledge, this is the first national study to characterize the long-term temporal, demographic, geographic, socioeconomic, and projected mortality patterns of lung cancer with documented sleep apnea in the United States.

The sustained increase observed in this study should be interpreted as an increase in deaths in which the two conditions were jointly documented, rather than as evidence that overall lung cancer mortality increased or that sleep apnea caused the fatal cancer. Nevertheless, the direction of the trend is consistent with a broader rise in US mortality involving sleep apnea and other chronic diseases. CDC WONDER studies have reported increasing mortality involving sleep apnea with cardiovascular disease, hypertension, obesity, and diabetes, frequently with disproportionate burdens among men, older adults, rural populations, and residents of the Midwest (18–20). Several processes may contribute simultaneously: population aging; increasing obesity, cardiometabolic disease, and multimorbidity; a rising pool of people living long enough to receive both cancer and sleep-apnea diagnoses; and improved recognition, testing, coding, and death-certificate documentation of sleep apnea. Because sleep apnea remains underdiagnosed and is infrequently listed on death certificates, the low absolute AAMR probably represents only the visible fraction of the true comorbidity burden. Conversely, improved ascertainment over time may account for part of the relative increase, particularly when the baseline rate is very low.

There is biological plausibility for OSA to contribute to an adverse clinical phenotype in lung cancer, but this evidence should be distinguished from the broader category of documented sleep apnea examined in the present study. Most mechanistic research has focused specifically on OSA and intermittent hypoxia. Chronic intermittent hypoxia can activate HIF-1α and VEGF signaling, oxidative stress, inflammatory pathways, angiogenesis, epithelial–mesenchymal transition, macrophage polarization, and immune escape, and experimental intermittent hypoxia has accelerated tumor growth and migration in lung-cancer models (21). Clinical studies have also linked nocturnal hypoxemia or OSA severity with lung cancer occurrence or outcomes (7, 8, 11, 12). However, the epidemiological literature remains heterogeneous, with some studies reporting no overall increase in cancer-related death or sex-specific and inverse associations (13, 22, 23). Importantly, these studies generally evaluated clinically characterized OSA, whereas the present study used ICD-10 code G47.3 from death certificates and therefore could not distinguish obstructive from central or unspecified sleep apnea. Accordingly, the OSA literature provides mechanistic and clinical context for interpreting our findings but should not be considered direct evidence that the mortality trends observed here are attributable specifically to OSA.

The marked sex and age differences require similar caution. Men had an AAMR three times that of women in the present study. This pattern may reflect the higher prevalence and severity of OSA in men, historical sex differences in tobacco and occupational exposures, differences in lung-cancer subtype and mortality, and greater likelihood that sleep apnea is clinically recognized and recorded in men. At the same time, sex-specific cohort studies indicate that the relationship between OSA and lung-cancer incidence may differ by underlying pulmonary disease and hypoxic susceptibility, with increased risks reported among women with COPD and hypoxia-related comorbidities (23). Thus, the male excess in death-certificate co-documentation should not be interpreted as evidence that women are biologically protected. Similarly, the concentration of deaths in adults aged 65 years or older reflects the convergence of lung-cancer incidence, OSA prevalence, multimorbidity, frailty, and reduced cardiopulmonary reserve at older ages. This predominance in absolute mortality is not inconsistent with prior work reporting a stronger relative association between OSA severity and cancer mortality in patients younger than 65 years (10); relative risk and population-level absolute burden address different questions.

The geographic pattern—higher mortality in the Midwest and West, a clear nonmetropolitan excess, and substantial variation among states with similar SDI values—suggests that broad socioeconomic development alone does not explain the observed burden. Comparable CDC WONDER studies have reported higher sleep-apnea-related cardiovascular and obesity mortality in rural areas and the Midwest (18). Potential contributors include regional variation in smoking, obesity, COPD, occupational exposures, healthcare-seeking behavior, availability of sleep laboratories and oncology specialists, transportation barriers, insurance coverage, and state-specific certification or ICD-coding practices. Several state-year observations showed positive frontier gaps relative to the empirical best-performance frontier. These observations are hypothesis-generating rather than rankings of healthcare performance because rare outcomes are sensitive to small numerators, suppressed cells, stochastic fluctuation, migration, and differences in documentation. Validation using cancer-registry incidence and survival data linked to sleep-study, claims, and electronic health-record information is needed before attributing state differences to clinical practice or policy.

The findings have practical implications for integrated care. In older adults with lung cancer—particularly men and patients with obesity, COPD, hypertension, cardiovascular disease, daytime sleepiness, witnessed apneas, or nocturnal hypoxemia—clinicians should maintain a low threshold for a structured sleep history and guideline-based evaluation for sleep apnea. Identifying and treating sleep apnea may improve symptoms, sleep quality, perioperative safety, and cardiometabolic health, all of which are relevant to treatment tolerance and quality of life. However, this study does not demonstrate that positive-airway-pressure therapy reduces lung-cancer progression or mortality, and treatment should not be presented as an anticancer intervention on the basis of these data.

This study has several strengths, including the use of a national multiple-cause-of-death database covering more than two decades and the application of Joinpoint, geographic, socioeconomic, frontier, and forecasting analyses. Several limitations should also be emphasized. First, death certificates are vulnerable to diagnostic and coding errors, and sleep apnea is likely to be substantially underreported. Second, ICD-10 code G47.3 includes obstructive, central, and unspecified sleep apnea. Although much of the available mechanistic and epidemiological literature concerns OSA, the present dataset does not permit subtype-specific identification; therefore, our findings should be interpreted as relating to documented sleep apnea broadly rather than OSA specifically. OSA-related evidence discussed in the Introduction and Discussion is provided as biological and clinical context and cannot be directly generalized to all cases captured by G47.3. Third, the database does not provide the number of living patients with both conditions; consequently, this study cannot estimate lung-cancer incidence, the prevalence of sleep apnea among patients with lung cancer, relative risk, or case-fatality. Fourth, individual-level information on smoking, BMI, alcohol use, COPD, cardiometabolic disease, sleep-apnea subtype or severity, hypoxic burden, positive-airway-pressure treatment, cancer histology, molecular alterations, stage, therapy, insurance, and healthcare access was unavailable. Residual confounding is therefore unavoidable. Fifth, the SDI analysis was ecological and based on annually matched state-level observations. Although the use of year-specific SDI values allowed temporal alignment between sociodemographic development and mortality from 1999 to 2020, state-level associations cannot be extrapolated to individuals. In addition, suppressed or unreliable mortality estimates for state-year observations with small numbers may have influenced the correlation and frontier analyses. Finally, the ARIMA model was estimated from only 22 annual observations, showed modest residual autocorrelation, and systematically overestimated observed AAMRs during 2021–2024; consequently, the 2021–2030 forecasts should be regarded as conditional and exploratory projections that may be sensitive to structural changes in mortality patterns, population structure, disease recognition, and death-certificate coding practices. These limitations reinforce that the results are descriptive and hypothesis-generating rather than causal.

## Conclusion

Mortality in which lung cancer was the underlying cause and sleep apnea was documented as a contributing condition increased markedly in the United States from 1999 to 2020 and was concentrated among older adults, men, White and non-Hispanic individuals, residents of the Midwest and West, and nonmetropolitan populations. Geographic heterogeneity and frontier gaps were not fully explained by state-level SDI. The historical trajectory also supported conditional, exploratory projections of further increases through 2030, although the model overestimated observed AAMRs during the 2021–2024 validation period. These findings identify a clinically complex population that may benefit from greater attention to sleep health within oncology and respiratory care. Because death-certificate data cannot establish causality or treatment effects, and because forecasting performance was limited, linked clinical datasets and prospective studies are needed to clarify mechanisms, distinguish true disease growth from improved documentation, and determine whether detection and treatment of sleep apnea can improve outcomes in patients with lung cancer.

## Data Availability

Publicly available datasets were analyzed in this study. The mortality data are available from CDC WONDER at https://wonder.cdc.gov/.
