# Supplementary material for: Uncovering the mortality burden of lung cancer with documented sleep apnea in the United States: a 22-year population-based study
Source: Front Public Health. 2026 Sep 30;14:1925388. doi: 10.3389/fpubh.2026.1925388 (PMC13661379; doi:10.3389/fpubh.2026.1925388)
Supplement: Supplementary file 1 [file Supplementary_file_1.docx]

Supplementary Figure 1. Flow diagram of study population selection.

Supplementary Figure 2. Age- and sex-specific mortality rates for lung cancer and documented sleep apnea-related deaths in 1999 and 2020.

Supplementary Figure 3. Age composition of reportable lung cancer and documented sleep apnea-related deaths, 1999–2020. For each year, percentages were calculated using the total number of deaths across age-by-sex cells with non-suppressed counts as the denominator. Because CDC WONDER suppresses small-count age-by-sex cells, the figure does not represent the complete annual age distribution of all deaths.

Supplementary Figure 4. Temporal trend in age-adjusted mortality rates for lung cancer and documented sleep apnea-related deaths, 1999–2020.

Supplementary Figure 5. Annual deaths and age-adjusted mortality rates for lung cancer and documented sleep apnea-related mortality, 1999–2020.

Supplementary Figure 6. Distribution of lung cancer and documented sleep apnea-related age-adjusted mortality burden across annually matched state-year Sociodemographic Index (SDI) values, 1999–2020.
